# Prophylactic and Therapeutic Usage of Drains in Gynecologic Oncology Procedures: A Comprehensive Review

**DOI:** 10.3390/jpm15060254

**Published:** 2025-06-16

**Authors:** Chrysoula Margioula-Siarkou, Aristarchos Almperis, Emmanouela-Aliki Almperi, Georgia Margioula-Siarkou, Stefanos Flindris, Nikoletta Daponte, Alexandros Daponte, Konstantinos Dinas, Stamatios Petousis

**Affiliations:** 1 Gynaecologic Oncology Unit, 2nd Department of Obstetrics and Gynaecology, Aristotle University of Thessaloniki, 54642 Thessaloniki, Greece; 2 Department of Obstetrics and Gynaecology, Medical School, University of Thessaly, 41500 Larissa, Greece

**Keywords:** drainage, gynecologic oncology, lymphadenectomy, hysterectomy, febrile morbidity

## Abstract

The use of post-operative drainage has been a topic of debate for several years. While the trend has increasingly shifted toward avoiding routine drainage, opinions on its necessity remain divided. The main objective of this comprehensive review is to effectively summarize and present the current knowledge and up-to-date evidence on the role of prophylactic drainage in women undergoing obstetric, oncological, or other types of gynecological surgical procedures in terms of the indications, post-operative surgical infections, morbidity recovery, post-operative complications and outcomes. Prophylactic drainage does not seem to decrease morbidity in cases of lymphadenectomy and radical hysterectomy. Debulking surgery does not necessitate prophylactic drainage in the majority of cases; however, its usage should be individualized based on the surgical complexity. Conflicting evidence exists regarding drains’ effectiveness in preventing anastomotic leakage, with high rates of re-operation and abscess formation noted. Despite the fact that vaginal drains may help with hematoma and infectious morbidity, the overall benefit of vaginal and peritoneal drains in preventing post-operative morbidity is questionable. Finally, negative pressure wound therapy may reduce surgical site infection rates in patients undergoing cytoreductive surgery for ovarian cancer. Although there is still a great need for further investigation, the topic has been covered adequately by many prospective trials and the international guidelines have provided clear suggestions to guide physicians in clinical practice. However, need for individualization and personalized strategies is well emphasized by the published evidence in an effort to balance the benefits and risks of drainage usage determined by the type of surgery and patient status.

## 1. Introduction

The use of post-operative drainage has been a topic of debate for several years [1]. While the trend has increasingly shifted toward avoiding routine drainage, opinions on its necessity remain divided. Proponents argue that drainage aids in the early detection of complications and prevents abscess formation by preventing the accumulation of blood and infected fluids [2]. It is also suggested that drainage might reduce post-operative pain by evacuating trapped gas, although this claim is contested. The frequency of drainage application varies among surgeons, with junior surgeons more likely to use drainage compared to their senior counterparts. Despite the perceived benefits, drainage is not without its drawbacks. Complications associated with drainage include an increased risk of incisional hernias, infections, prolonged operation times, extended hospital stays, and post-operative pain [3]. Drainage can also cause intra-operative intestinal injury, post-operative fever, pain at the drainage site, and issues such as blockage, dislodging, and kinking of the drain [4]. Consequently, it is recommended that drains be removed as soon as possible after surgery.

Regarding the current status of drainage usage, in the 2018 guidelines for the prevention of surgical site infections (SSIs), the World Health Organization (WHO) endorsed the utilization of negative pressure wound therapy (NPWT) for adult patients with closed surgical incisions in situations deemed high risk [5]. The use of drains following radical hysterectomy and pelvic lymph node dissection remains controversial. Some systematic reviews suggest that drains reduce lymphocyst formation after pelvic lymphadenectomy, while others highlight the benefits of early mobilization associated with the absence of drains, including reduced rates of post-operative ileus and surgical site infection and shorter hospital stays [6,7]. The absence of solid evidence and the mainly retrospective character of the data cannot justify routine drain use but may indicate selective use in high-risk patients in terms of the comorbidities, surgery duration or intra-operative complications.

The main objective of this comprehensive review is to effectively summarize and present the current knowledge and up-to-date evidence on the role of prophylactic drainage in women undergoing obstetric, oncological, or other types of gynecological surgical procedures in terms of the indications, post-operative surgical infections, morbidity recovery, post-operative complications and outcomes, with a special effort made to underline the need for personalized management in these cases.

## 2. Materials and Methods

The aim of the present comprehensive review was to evaluate the existing literature, including guidelines published by internationally acknowledged medical organizations, colleges, associations, societies, committees, and study groups regarding the post-operative usage of drainage in various gynecologic oncology procedures. This comprehensive review was conducted following the preferred reporting items for systematic reviews and meta-analysis (PRISMA) recommendations to critically evaluate the current literature and conduct a narrative and up-to-date review. The PubMed, Scopus and Embase electronic databases were searched up to July 2024 for relevant articles published in the period 2014–2024. The search strategy was formed by combining the MeSH terms and keywords ‘Drainage AND cytoreduction’, ‘Drainage AND hysterectomy’, ‘Drainage AND lymphadenectomy’, ‘Drainage AND lymphadenectomy AND vulvar cancer’, ‘Drainage AND debulking’, and ‘Drainage AND gynecologic oncology’. Prospective randomized clinical trials (RCTs), prospective observational studies and meta-analyses written in the English language were set in the center of interest for interpretation of their results. Retrospective studies, in the absence of higher-level evidence, were also considered for the purpose of the present review. All the studies identified from the search strategy were imported into a reference management software (Zotero 6.0.30) for the elimination of duplicate data and further assessment. The systematic search revealed 339 items with potential for inclusion in our narrative review. 

All the identified studies were screened by two of the authors based on their full-text manuscripts, while articles irrelevant to the objective of the present study were excluded. The eligibility of the retrieved articles was independently determined by 2 reviewers (C.M-S., A.A.). The main outcomes set in the center of interest were the wound infection rate and wound complications rate, incidence of vault hematomas in cases of hysterectomy, febrile morbidity rate, length of hospital stay, readmission, incidence of pelvic cellulitis and lymphocyst formation percentages in cases of lymphadenectomy. 

**Figure 1:**
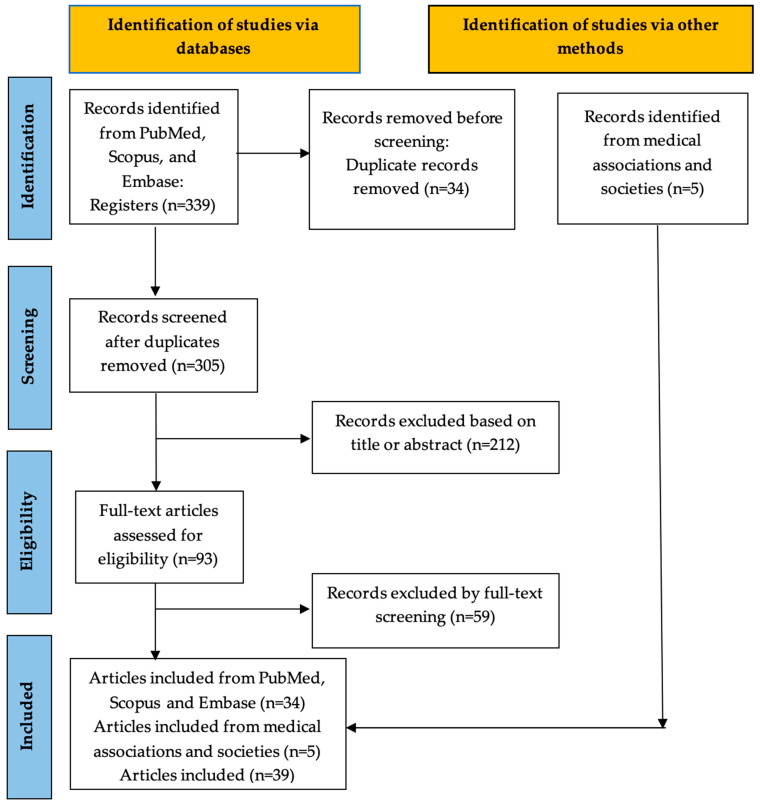
Flowchart of the study selection.

There were finally 39 published studies and society guidelines/recommendations regarding the post-operative usage of drainage in various gynecologic oncology procedures included in the present review. The flowchart of the study selection is presented in Figure 1.

## 3. Drainage of Subcutaneous Wounds After Cytoreduction for Ovarian Cancer

The topic of intra-operative placement of subcutaneous wound drains has been extensively covered as a method to reduce wound complications. This technique is advantageous for the removal of fluid accumulated in subcutaneous tissue and helps in reducing dead space. Regarding non-obstetric patients, the role of negative pressure wound drainage showed conflicting results in various gynecologic surgeries [8,9,10]. However, the inconsistent results may be attributed to the heterogeneity of the population and that of the procedures. Extensive cytoreductive surgery, despite its lengthy duration, is crucial for reducing intraperitoneal residual tumors in advanced or recurrent ovarian cancer. In gynecologic cancers requiring additional treatments—like adjuvant chemotherapy after cytoreductive surgery for ovarian cancer or radiotherapy and/or chemotherapy following radical hysterectomy for cervical cancer—wound complications have a crucial impact on disease progression by delaying the scheduled treatment. 

Regarding cytoreduction surgery for ovarian cancer, an inadequate number of published studies are available, and the few data are derived mainly from retrospective cohorts, providing low-quality evidence. A retrospective cohort study by Kim et al. encompassed patients who underwent cytoreductive surgery for epithelial ovarian cancer. The wound infection rate and the wound complications rate between groups using and not using subcutaneous wound drains were compared and a reduction rate of up to 80% and up to 65%, respectively, was observed [11]. Another more recent retrospective cohort, examining 312 patients undergoing surgery with a midline incision for ovarian cancer, suggested a roughly 60% lower wound complication risk and a lower rate of seroma formation (6.1% vs. 16.0%; *p* = 0.015) when using subcutaneous negative pressure drains compared to standard dressing [12]. Although both studies identified the placement of wound drain as an independent prognostic factor for uncomplicated wound outcomes, no statistically significant difference between the two arms regarding progression-free and overall survival was observed. 

One of the limitations of the above-mentioned studies, which was the selection bias of including only cases with ovarian cancer, was addressed in another retrospective cohort study. In this study, the benefit of subcutaneous negative pressure drainage without subcutaneous suture on wound patients who underwent abdominal surgery was set in the center of analysis. The laparotomy indication included both malignant and benign gynecological conditions. It was observed that a statistically significantly higher rate of clear healing was achieved in the drainage arm only in the subgroup of malignant cases (95.6% vs. 82.8% (*p* = 0.032)) [13].

However, the KGOG-4001, the first gynecologic-specific RCT of 162 females undergoing midline laparotomy, found recently that adding a subcutaneous closed-suction drain neither lowered neither the 4-week nor cumulative wound dehiscence, nor the SSI rate. Suturing the extra fat layer was the only protective step [8].

To summarize, there are two retrospective studies [11,12] with concordant outcomes standing in favor of the potential beneficial role of negative pressure wound therapy in cytoreduction surgery for ovarian cancer. The only high-level evidence available for gynecologic patients indicates no routine use of subcutaneous drainage after midline laparotomy. A basic remark on the earlier published evidence is the fact that, despite the possible benefit of subcutaneous drainage to prevent post-operative complications (modest seroma and infection rate reductions), the final decision should be personalized based on individualized parameters, such as obesity, hypertension, diabetes and other medical history [8,11,12,13]. However, the lack of adequate randomized trials or prospective studies leaves enough space for debate and further study performance, mainly focused on highlighting personalized strategies and indications for drainage placement.

## 4. Drainage Following Vaginal or Laparoscopic Hysterectomy

Hysterectomy remains the most common non-obstetric procedure among female patients. Despite the rising rate of laparoscopically and robotically assisted hysterectomies, the vast majority are still performed abdominally [14,15,16]. All hysterectomies, regardless of the surgical approach, require the vault to be opened. Therefore, inserting a drain vaginally might help in avoiding the morbidity associated with the usage of abdominal drainage in an effort to reduce the post-operative morbidity related to the development of hematomas. The vaginal route of hysterectomy is associated with a higher incidence of vault hematomas than abdominal hysterectomies. 

In the literature, the hematoma rates can vary widely, being reported to be as high as 98%. However, in clinical practice, the incidence of infected hematomas may be as low as 10% due to differing circumstances and study methodologies [17]. Vault hematomas are significant due to the post-operative morbidity, including infection, pelvic pain, and prolonged hospital stays. 

Krishnaswamy et al. [18] have conducted a systematic review and meta-analysis of ten studies involving 1778 women to study the potential benefit of vaginal drain usage. The use of a vaginal drain after hysterectomy was indicated to significantly reduce the incidence of vault hematoma (OR 0.22, 95% CI 0.08–0.57) and febrile morbidity (OR 0.54, 95% CI 0.40 to 0.73). However, no statistically significant reduction in antibiotics usage and length of the hospital stay was observed. The types of drains used in the included studies were a large-bore Foley catheter, a Robinson’s drain, a T-tube suction drain and a Jackson–Pratt closed-suction drain, while the type of hysterectomy included all potential approaches. The results of the meta-analysis were not confirmed by a consequent prospective randomized study comparing drainage in the peritoneal cavity and cul-de-sac after vaginal hysterectomy with no drainage usage. No statistically significant difference was observed in terms of the infectious morbidity and post-operative complications [19].

All of the above-mentioned studies export their data from patients undergoing laparoscopically assisted vaginal hysterectomy or vaginal and abdominal hysterectomy. There has been only a retrospective cohort study performed by Oh et al. enrolling 504 patients undergoing laparoscopic hysterectomy, in which pelvic drainage placement abdominally or vaginally was compared in terms of the post-operative fever, readmission and re-operation rates [20]. No significant difference was indicated between the two groups. Therefore, the authors concluded that vaginal vault placement of a closed pelvic gravity drain may represent a promising new approach for reducing infectious complications associated with pelvic hematomas, emphasizing the need to personalize the approach based on the patient and special surgical requirements. The efficacy of this minimally invasive technique warrants further investigation through larger-scale clinical trials since no other study has compared the adequacy between vaginal or abdominal drains in laparoscopic hysterectomy.

Unfortunately, there are no published data available referring to the role of drainage in cases of hysterectomy combined with sentinel lymph node biopsy for malignant conditions. However, taking into consideration the accumulated clinical experience, drainage usage offers no further advantage, since the vast majority of patients experience no severe post-operative complications after laparoscopic hysterectomy, undergo quick mobilization and a short duration of stay and are discharged within 24 to 48 h after the surgery.

In conclusion, there has been limited high-quality evidence published to stand in favor of the potential benefit of vaginal drain placement independently of the hysterectomy type. However, no definitive conclusions have yet been deduced. To further explore this potential, prospective comparative clinical trials are warranted. In any case, placement of a vaginal drain has certain scientific merit and allows an individualized approach to cases, taking into consideration the type of surgery, duration of surgery and special patient characteristics [18,19,20].

## 5. Drainage Following Radical Hysterectomy and Lymphadenectomy or Lymphadenectomy for Various Gynecological Malignancies (Pelvic and/or Para-Aortic)

Over the past 30 years, the potential effectiveness of drainage in radical hysterectomy followed by pelvic lymphadenectomy lay in the prevention of post-operative febrile morbidity and formation of lymphocysts or fistulas. However, the utilization of antibiotics and the surgical tendency to leave the retroperitoneum open and thus enable the reabsorption of the lymph fluid from the abdominal cavity have made drainage quite debatable [21]. Jensen et al., in a retrospective series of 67 patients undergoing radical hysterectomy and bilateral pelvic lymphadenectomy (RHPL) for early-stage cervical cancer with drainage, found no significant improvement in terms of the febrile morbidity rates, incidence of pelvic cellulitis and length of hospital stay compared to the group without drainage. In fact, on some occasions, drainage contributed to post-operative complications, such as local infection or cellulitis at the exit site of the drain, prolonging the use of antibiotic therapy [21]. Consistent with these data were the results of two prospective randomized studies [22,23] and one prospective non-randomized study [24] monitoring a similar population of cervical cancer patients undergoing RHPL with homogenous epidemiological, clinical, and surgical characteristics, focusing on the post-operative morbidity, complications and lymphocyst formation. Evaluation of possible lymphocysts was performed clinically with a CT scan or the use of transabdominal/vaginally sonography. No statistically significant difference favoring drainage usage in the reduction of the infection, fistula or lymphocyst formation rates was observed in any of the above studies. In fact, lymphocysts were observed only in patients with closed-suction drainage, possible due to the presence of the foreign body, as Jensen et al. first noticed. 

The most up-to-date Cochrane review included four studies, setting in the center of focus patients who underwent systematic pelvic or pelvic and para-aortic lymphadenectomy for various gynecologic malignancies, regardless of the surgical approach [6]. It was concluded that the overall rates of lymphocyst formation were comparable independently of the type of drainage. A sub-group analysis indicated that a trend for a higher incidence of lymphocyst formation was detected in the drainage group in cases where the peritoneum was left open. As a result, the potential advantage of drainage placement in the prevention of pelvic infection or lymphocyst formation was not justified. Similar results have also been observed in a prospective non-randomized study of 143 oncological female patients published by Bafna et al. [25]. Moreover, Morice et al. have studied the potential role of drainage in the lymphocyst formation after complete para-aortic lymphadenectomy up to the level of the left renal vein for ovarian or cervical carcinoma [26]. Drainage was significantly associated with a higher rate of cyst formation, post-operative complications and a longer hospital stay.

As an overall conclusion, high-quality data derived mostly from prospective studies have clearly suggested that the practice of drainage does not generally improve the risk of post-operative morbidity in women undergoing RHPL. Lymphocyst formation is a common complication after pelvic or para-aortic lymph node dissection, potentially causing lower limb edema, deep vein thrombosis, secondary infection, pain or ureteral obstruction [27]. Taking into consideration the open retroperitoneal space and the usage of prophylactic antibiotics, drainage could be personalized and used only on high-risk occasions, especially in the presence of intra-operative retroperitoneal bleeding, oozing or ureteral injury. A summary of the above-mentioned results is displayed in Table 1. 

## 6. Drainage Following Complex Debulking with Colectomy and Peritonectomy

Another issue of debate is the potential value of drainage in monitoring the potential anastomotic leakage after a modified posterior pelvic exenteration (MPPE). Most patients with advanced primary ovarian cancer are treated with cytoreductive surgery and platinum-based chemotherapy. Achieving a macroscopic complete resection during surgery significantly improves survival rates [28]. Due to the rectosigmoid proximity to the female pelvic organs and its frequent involvement in ovarian cancer, rectosigmoid resection is commonly performed. En bloc resection of pelvic tumors, including the uterus and rectosigmoid (known as modified posterior pelvic exenteration), is a valuable part of cytoreductive surgery. Despite advances in techniques, anastomotic leakage remains a serious complication, with an incidence ranging from 0.8% to 10% in these procedures [29,30]. To reduce this risk, surgeons have explored various preventative measures, including the use of a transanal drainage tube (TDT) in rectal cancer surgeries to prevent leakage after a low anterior resection. As is indicated from a recent retrospective review concerning patients who had undergone an MPPE for primary ovarian, tubal, or peritoneal cancer, TDT placement seems to be an effective and safe way to decrease the rate of anastomotic leakage and the need for a diverting stoma. However, some patients will inevitably require a diverting stoma in addition to TDT placement [31].

Several randomized prospective studies in Western countries have found prophylactic abdominal drainage tubes to be unnecessary [32,33]. A Japanese retrospective study involving 260 patients who underwent colectomy and suprapromontory anastomosis showed that, when comparing 124 patients with and 136 patients without prophylactic drainage tubes, no statistically significant difference was observed in terms of the post-operative complications or abscess formation. On the contrary, there was a significantly higher incidence of re-operation in the drained group. The study concluded that prophylactic drainage tubes are unnecessary even in cases involving extensive resection and regional lymphadenectomy and no crucial benefit can be proven [34].

A recently published comprehensive review of the literature on the use of prophylactic drainage in a general surgical setting shows wide variability. Through systematic reviews and meta-analyses, it was consistently showed that in many gastrointestinal, hepatobiliary and colorectal procedures, drains do not lower the rates of abscess, anastomotic leak or mortality and may raise the wound infection risk. Routine drainage is frequently linked to a longer hospital stay, higher wound complications and patient discomfort [35].

In summary, it seems that drainage placement after extensive cytoreductive surgery involving posterior pelvic exenteration not only does not lead to a decrease in post-operative complications but might also be associated with increased risk of re-operation. However, as also mentioned in the ESGO guidelines, personalized consideration of drainage placement is an option, as the benefit–risk ratio may be balanced based on the individualized parameters of every case, mainly the duration of surgery, patient ECOG status, medical comorbidities and radicality of surgical procedures [36].

## 7. Drainage Following Inguinofemoral Lymphadenectomy in Vulvar Cancer

Vulvar cancer represents a challenging gynecologic malignancy with unique surgical considerations. For early-stage vulvar cancer, management requires surgically removing the tumor with wide local excision along with sentinel lymph node (SLN) biopsy for unifocal tumors < 4 cm without suspicious inguinofemoral lymph nodes on clinical examination and imaging, or inguinofemoral lymphadenectomy (IFL) for tumors ≥ 4 cm and/or in case of multifocal invasive disease [37]. In clinical practice, more and more focus is dedicated to the drainage technique after IFL due to its impact on the post-operative complications. 

The MAMBO study is a Dutch nationwide prospective trial comparing volume-controlled drainage removal (MAMBO-IA, when <30 mL/24 h) with the fixed period short drainage protocol (MAMBO-IB, removal at five days) in 251 vulvar cancer patients undergoing IFL. It was conducted across eight oncology centers. It specifically compares volume-controlled drainage (MAMBO-IA, <30 mL/24 h) and a fixed period short drainage protocol (MAMBO-IB, five days) in vulvar cancer patients undergoing IFL. This study found that volume-controlled drainage significantly reduced the lymphocele formation rate (46% vs. 75%, 95% CI 8–49, *p* = 0.006) and the incidence of at least one complication by 29% [38]. Gould et al. found that the duration of surgical drainage was not an independent predictor of infection or post-operative complications such as lymphedema [39]. The data from two retrospective studies are contradictory, with Walker et al. suggesting that a longer duration of drainage is linked to higher rates of lymphedema [40]. On the contrary, Pontre et al. found that drainage is associated with a higher incidence of post-operative groin cellulitis and no difference in lymphocyst formation, wound infection, duration of hospital stay or lower limb lymphedema [41]. The current literature is vague and no secure conclusion can be deduced. Important limitations are the data that derive from retrospective cohorts with heterogenous and small samples. Although in clinical practice drainage after IFL is a common procedure, only one high-level study supports the beneficial role of volume-controlled drainage regarding the lymphocele formation rate. However, even after that, the incidence of complications remains considerable, underscoring the need for larger, prospective trials investigating additional factors.

## 8. Guidelines

International guidelines approaching the issue of perioperative management regarding the placement of drains, in an effort to minimize the rate of post-operative complications and shorten the recovery time, have currently offered clear suggestions. According to ACOG COMMITTEE OPINION number 750, which was reaffirmed in 2024 [42], and the ERAS (enhanced recovery after surgery) protocol, surgical drains and vaginal packs should be avoided or taken out as early as possible post-surgery. Relatively, the habitual use of nasogastric, abdominal, and vaginal drains limits mobility, elevates morbidity, and extends hospital stays, with minimal evidence of their benefit. Avoiding regular use of tubes is strongly recommended to increase mobility and shorten the hospital stay, since its use is poorly associated with a lower rate of anastomotic leaks but frequently with pulmonary complications. The 2019 updated ERAS protocol [43] reaffirmed that there is no solid evidence of the routine use of subcutaneous or peritoneal drains reducing SSIs, since early biofilm colonization can occur. It is concluded that individualized, procedure-specific drain placement is preferable and drains should preferably be taken out in the first 24 h after surgery. Furthermore, the European Society of Gynecological Oncology mainly focuses on the prevention and management of post-operative complications, such as intra-abdominal and collections and abscesses [36]. Diaphragmatic surgery does not justify routine chest tube placement, unless there are high-risk characteristics or pre-operative pleural effusion (grade B). In cases of clinically significant post-operative collections or abscesses, imaged-guided percutaneous drainage is recommended (grade B), which in cases of ascites and lymphadenectomy is proposed just as an option to consider (grade C). Nasogastric intubation heightens patient discomfort and the post-operative respiratory infection risk following elective abdominal surgery, without lowering the wound dehiscence or anastomotic leak rates. Likewise, routine peritoneal drainage confers no benefit after bowel resection for ovarian cancer [44,45]. In summary, according to the major international guidelines, the prudent and selective implementation of surgical drains and tubes is suggested only where it is needed. High-risk situations include uncontrollable/diffuse oozing, expected major lymphatic leakage, or technically tenuous bowel anastomoses [36,42,43]. The early removal is the appropriate approach, since the benefit of its routine use cannot be justified by the existing published data. Contrary to the guidelines and despite the well-known related adverse events, both the nasogastric tubes and the peritoneal drains continue to be widely adopted (reported rates of 56% and 52–75%, respectively) [44]. The European ERAS implementation survey 2023 by Gómez-Hidalgo et al. confirms the lack of clinical implementation, with only 25% of the centers ‘normally/always’ avoiding drains after laparotomy [45]. However, as thoroughly stated in the literature, a personalized approach should always be considered based on the specifical medical and surgical parameters of cases. A summary of the guidelines regarding the usage of tubes peri- and post-operatively is presented in Table 2.

## 9. Conclusions and Future Directions

**Figure 2:**
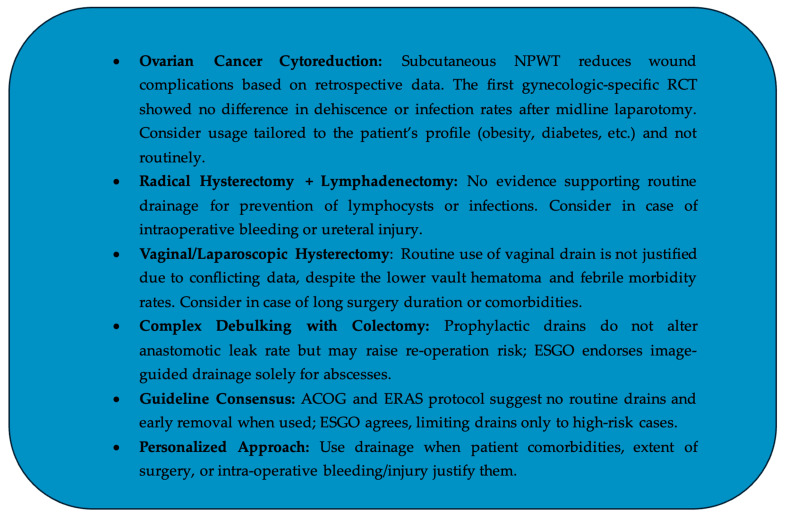
Key insights.

The present article discusses the efficacy and necessity of using drains in post-operative care, particularly in gynecologic oncology surgeries. It is suggested that negative pressure wound therapy may reduce the surgical site infection rates in patients undergoing cytoreductive surgery for ovarian cancer. It is highlighted that while vaginal drains may help with hematoma and infectious morbidity, the overall benefit of vaginal and peritoneal drains in preventing post-operative morbidity is questionable. Specifically, drains do not appear to improve the post-operative outcomes in women undergoing radical hysterectomy with pelvic lymphadenectomy, but they could still be reserved for high-risk situations, such as intra-operative bleeding or injury. However, their routine use may not be necessary and could lead to complications, particularly in cancerous patients with a history of chemotherapy or radiotherapy. Conflicting evidence exists regarding drains’ effectiveness in preventing anastomotic leakage, with high rates of re-operation and abscess formation noted. In any case, despite the rather non-supportive data on drainage placement for various types of surgeries for gynecologic oncology reasons, the final decision should be based on thorough consideration of individualized parameters, such as the radicality and duration of surgery, patient status, existence of obesity, diabetes and immunosuppression, in an effort to set the strategy for drainage placement in the overall framework of modern personalized medicine. The key insights are displayed in Figure 2.

Abbreviations: SSIs, surgical site infections; NPWT, negative pressure wound therapy; RHPL, radical hysterectomy and bilateral pelvic lymphadenectomy; MPPE, modified posterior pelvic exenteration; TDT, transanal drainage tube; SLN, sentinel lymph node; IFL, inguinofemoral lymphadenectomy.

## Figures and Tables

**Table 1:** Drainage following radical hysterectomy and lymphadenectomy or lymphadenectomy for various gynecological malignancies. ^1^ RHPL: radical hysterectomy and bilateral pelvic lymphadenectomy, ^2^ NS: not statistically different, ^3^ LND: lymphadenectomy.

Studies	Type of Study	Patients	Type of Operation	Type of Drain	Febrile Morbidity Rates (Drain vs. No Drain)	Mean Length of Hospital Stay in Days (Drain vs. No Drain)	Lymphocysts Formation (Drain vs. No Drain)	Post-Operative Complications (Drain vs. No Drain)	Conclusions Regarding the Prophylactic Drainage
Jensen et al. [21]	Retrospective cohort	Early-stage cervical cancer	^1^ RHPL	Jackson–Pratt closed-suction drainage	32.8% vs. 29.1% (^2^ NS)	7.6 ± 2.4 vs. 7.0 ± 1.3 (^2^ NS)	-	-	Possibly unnecessary with post-operative complication risk
Srisomboon et al. [22]	Prospective randomized	Early-stage cervical cancer	^1^ RHPL	Retroperitoneal low-pressure closed-suction drains	5.8% vs. 0% (^2^ NS)	9.4 ± 1.6 vs. 9.2 ± 1.4 (^2^ NS)	^2^ NS, *p* = 0.2	-	Can safely be omitted
Franchi et al. [23]	Prospective randomized	Early-stage cervical cancer	^1^ RHPL	Passive or active suction drains	-	-	5.9% vs. 0.9% (^2^ NS, *p* = 0.06)	0.53% vs. 0.66% (^2^ NS)	Can safely be omitted in minimal intra-operative bleeding
Patsner et al. [24]	Prospective non-randomized study	Early-stage cervical cancer	^1^ RHPL	Jackson–Pratt closed-suction drainage	10% vs. 3.3% (^2^ NS)	5.5 vs. 4.5 (^2^ NS)	11.6% vs. 0% (^2^ NS)	-	Can safely be omitted
Bafna et al. [25]	Prospective non-randomized	Various gynecologic malignancies	Pelvic ± aortocaval ^3^ LND	Closed-suction retroperitoneal pelvic drainage	-	10 vs. 10 (^2^ NS)	7.2% vs. 2.7% (^2^ NS, *p* > 0.05)	-	No benefit over open peritoneum without drainage
Morice et al. [26]	Randomized trial	Ovarian or cervical carcinoma	Complete para-aortic ^3^ LND up to the level of the left renal vein	Pelvic suction drains (Bellovac; Astratech)	-	11 vs. 9 (*p* < 0.03)	5% vs. 24% (*p* < 0.05)	36% vs. 13% (*p* < 0.02)	Should be abandoned due to increased morbidity and hospitalization duration
Charoenkwan et al. [6]	Systematic review	Various gynecologic malignancies	Systematic pelvic or pelvic and aortic ^3^ LND	Passive or active suction retroperitoneal drains	-	-	^2^ NS	-	No benefit preventing lymphocyst formation

**Table 2:** Summary of guidelines regarding usage of tubes peri- and post-operatively.

International Guidelines	Upper Abdominal Complications	Diaphragmatic Surgery	Pleural Effusion	Post-Op Collections or Abscess	Diet	Urinary Catheter
ESGO guidelines [36]	Could be considered in large-volume ascites and extensive peritoneal and/or lymph node resections (III, C)	Not routinely indicated (III, B)	Could be considered in cases of high-volume pre-operative pleura effusion, frailty, hypoalbuminemia, and large diaphragmatic resection (III, B)	Preferable management: Image-guided percutaneous drainage (III, B)	-	-
ACOG committee opinion and ERAS protocol [42]	Avoidance of drains and vaginal packs				-No nasogastric tube -Regular diet and gum chewing 4 h post-operatively	Removal within 24 h
ERAS Society recommendations—2019 update [43]	Avoidance of drains/tubes					Post-operatively for a short period; preferably <24 h post-op

## Data Availability

Not applicable.
